# Probiotics as a Barrier Against *Salmonella enterica* and *Escherichia coli* in Contaminated Rice-Protein-Based Infant Formulas

**DOI:** 10.3390/foods15193477

**Published:** 2026-09-28

**Authors:** José Antonio Moreno-Muñoz, Pau Garcia-Sanz, Lucía Tadeo-Orellana, Helena Torrell, Adrià Cereto-Massagué, Carla Tort-Miró, June Sales-Flores, Manel Martín-Palomas, Jesús Jimenez

**Affiliations:** 1 Laboratorios Ordesa, Scientific Department, Parc Científic Barcelona, Baldiri Reixac 15-21, 08028 Barcelona, Spain; 01paugarcia@gmail.com (P.G.-S.); carlatort4@gmail.com (C.T.-M.); junesales14@gmail.com (J.S.-F.); manel.martin@ordesalab.com (M.M.-P.); jesus.jimenez@ordesalab.com (J.J.); 2 Biochemistry and Biotechnology Department, Universitat Rovira i Virgilli, Campus Sescelades C/Marcel·lí Domingo, 1, 43007 Tarragona, Spain; 3 Joint Unit Universitat Rovira i Virgili-Eurecat, Centre for Omic Sciences (COS), Eurecat, Centre Tecnològic de Catalunya and Unique Scientific and Technical Infrastructures (ICTS), Av. de la Universitat, 1, 43204 Reus, Tarragona, Spain; lucia.tadeo@eurecat.org (L.T.-O.); helena.torrell@eurecat.org (H.T.); adria.cereto@ce.eurecat.org (A.C.-M.)

**Keywords:** infant formula, probiotics, *Lacticaseibacillus rhamnosus* HN001, *Bifidobacterium infantis* IM-1, foodborne pathogens, *Escherichia coli*, *Salmonella enterica*, pathogen suppression, microbiota modulation, 16S rRNA sequencing

## Abstract

**Background/Aims:** The presence of pathogens such as *Salmonella enterica* and *Escherichia coli* in infant formulas, even at concentrations below the detection threshold, poses a health risk. Probiotics have been proposed as a strategy to limit pathogen proliferation. This study evaluated two probiotic strains (*Lacticaseibacillus rhamnosus* HN001 and *Bifidobacterium longum* subsp. *infantis* IM-1) to inhibit the growth of these pathogens under simulated conditions. **Methods:** Assays were conducted using reconstituted infant formula supplemented or not with the probiotics combination and challenged with *E. coli* or *Salmonella enterica* at low inoculum levels (1 or 10 CFU/10 g). A total of 112 samples were analyzed across eight trials, with two time points (T0h and T18h). Microbial composition was characterized by sequencing the V4 region of the 16S rRNA gene followed by bioinformatic analysis using QIIME2 and the SILVA database. **Results:** In the absence of probiotics, *E. coli* and *Salmonella enterica* showed a marked increase in relative abundance under the tested conditions (samples C1 and C10), reaching high relative abundances at T18h. In contrast, formulas supplemented with *Bifidobacterium longum* subsp. *infantis* IM-1 and *Lacticaseibacillus rhamnosus* HN001 were associated with markedly lower pathogen relative abundances of both pathogens, which were either undetectable or reduced to <1% in most conditions. A concurrent increase in beneficial bacterial genera such as *Lacticaseibacillus* and *Lactobacillus* was observed, indicating competition for ecological niches and nutrients. **Conclusions:** The probiotics *Bifidobacterium longum* subsp. *infantis* IM-1 and *Lacticaseibacillus rhamnosus* HN001 exerted a reduced proliferation effect against *E. coli* and *Salmonella enterica* in infant formulas contaminated at low inoculum levels, limiting pathogen proliferation and promoting the stability of beneficial microbiota. These findings suggest that incorporating probiotics may limit pathogen proliferation in infant formulas under the experimental conditions evaluated.

## 1. Introduction

Breast milk is universally recognized as the ideal form of nutrition for infants, providing all the necessary nutrients for growth along with infection-protective elements such as cytokines, antibodies, and oligosaccharides. When breastfeeding is not feasible, powdered infant formula (PIF) serves as the primary substitute; however, formula-fed infants lack the same level of immunological protection [1,2,3]. This is of critical importance because newborns, particularly preterm and low-birth-weight infants, possess immature immune systems and permeable gastrointestinal tracts, making them highly susceptible to severe foodborne infections [2,3,4].

Crucially, PIF is not a sterile product and is susceptible to both inherent and acquired microbial contamination [2,5,6]. Pathogens such as *Cronobacter sakazakii* and *Salmonella enterica* represent a major public health concern; they are classified as Category A hazards due to strong epidemiological and microbiological evidence linking them to PIF-mediated outbreaks [4,7,8]. Recent major incidents, including multi-country outbreaks of *Salmonella Poona* and *Salmonella Agona*, highlight the risk of persistent contamination within manufacturing facilities [9,10]. Category B includes pathogens such as *Klebsiella pneumoniae* and *Escherichia coli*, which are suspected but not definitively linked; and Category C (e.g., *Bacillus cereus*, *Clostridium botulinum*) cause illness but are rarely identified as formula-borne contaminants. *Escherichia coli* also represents a potential risk for systemic infections such as sepsis [2,4]. A significant challenge in ensuring safety is that even low-level contamination, often below the threshold of standard detection methods, can trigger extensive outbreaks upon the reconstitution and proliferation of the bacteria [4,9,10]. To mitigate these risks, the World Health Organization (WHO) recommends that PIF intended for infants aged 0–6 months be reconstituted with water at temperatures no lower than 70 °C to ensure the inactivation of pathogen vegetative cells [1,3,5,11]. Despite the establishment of requirements mandating that infant formula preparation instructions be included on packaging, several studies have shown that a very high percentage of parents/caregivers do not follow these recommendations and do not prepare their children’s bottles correctly, often using water at lower temperatures or failing to follow proper hygiene and sterilization protocols, even though the instructions or recommendations are printed on the formula packaging [1,5,12,13,14,15]. There is a scientific controversy regarding the optimal preparation temperature: while 70 °C is the standard recommendation, some evidence suggests that water at this temperature cools too rapidly during mixing to be lethal to pathogens, leading some researchers to propose temperatures exceeding 85 °C [5,16]. Approximately 84.1% of domestic formula-preparation machines and 21.7% of kettles fail to reach the 70 °C temperature recommended by the World Health Organization (WHO). This fact is primarily attributed to a combination of thermal physics, manufacturer design intent, and conflicting safety priorities. Research indicates that these machines are significantly less likely to achieve safe temperatures compared to traditional kettles, with one study finding that only 14.9% of machines produced water at 70 °C. The principal reasons for this discrepancy include the low-volume dispensing method inherent to many machine designs, which promotes rapid cooling and, together with a preference for preserving nutrients and probiotic viability over thermal decontamination, results in many feeds being prepared at unsafe temperatures. Up to 73% of caregivers do not wash their hands before preparation, and 30% do not read safety instructions. Overconfidence leads 84% of parents to rely on personal experience rather than official guidelines. These practices increase the risk of infants developing infections because of improper bottle preparation [4,5].

Conversely, concerns persist that such high heat may degrade heat-sensitive nutrients or reduce the viability of beneficial probiotics added to the formula [3,5,17].

In this context, the addition of probiotics, specifically *Lacticaseibacillus* and *Bifidobacterium* strains, is proposed as a strategy to inhibit pathogenic adherence and colonization through competition and the production of antimicrobial metabolites [3,17]. This study proposes the use of probiotics as “technological adjuvants” to create a biological barrier that limits pathogen proliferation when formulas are reconstituted at suboptimal temperatures (<70 °C) or when parents/caregivers do not prepare infant bottles correctly. The study does not propose the use of probiotics as an alternative to established hygienic practices for the preparation of powdered infant formula (PIF). In this study, we evaluated the ability of two probiotic strains (*Lacticaseibacillus rhamnosus* HN001 and *Bifidobacterium longum* subsp. *infantis* IM-1) to inhibit the proliferation of pathogens, *Salmonella enterica* and *Escherichia coli*, under simulated contamination scenarios. This study is highly relevant as it addresses the need for biological strategies to limit the growth of pathogens that remain hazardous even at concentrations below detection limits in infant formula [4,10]. Our conclusions indicate that these probiotic strains reduced the proliferation of target pathogens in reconstituted formula and could improve the intrinsic safety of infant formula [3,17].

## 2. Materials and Methods

### 2.1. Probiotic Strains and Preparation

The probiotics used in this study were *Bifidobacterium longum* subsp. *infantis* CECT 7210/*Bifidobacterium longum* subsp. *infantis* IM-1 and *Lacticaseibacillus rhamnosus* HN001. For bacterial preparation, HN001 strain was cultured in de Man, Rogosa and Sharpe (MRS) broth (Merck KGaA, Darmstadt, Germany) under anaerobic conditions at 37 °C for 24 h in an anaerobic DWS MC500 Workstation (Don Whitley Scientific, Bingley, UK), and the IM-1 strain was cultured in de Man, Rogosa and Sharpe (MRS) broth (Merck KGaA, Darmstadt, Germany) with 0.05% (*w*/*v*) of HCl-Cysteine (VWR International, Leuven, Belgium) under anaerobic conditions at 37 °C for 24 h in an anaerobic DWS MC500 Workstation (Don Whitley Scientific, Bingley, UK). Bacterial cells were harvested by centrifugation at 12,000× *g* for 10 min, washed three times with phosphate-buffered saline (PBS), and resuspended in sterile PBS. The bacterial concentration was adjusted to approximately 1 × 10^9^ CFU/mL using a turbidimeter (Grant Instruments, Cambridge, Royston, UK). Viable counts of the bacterial cell suspension were reconfirmed on plates of MRS agar for HN001 or MRS supplemented with 0.05% (*w*/*v*) HCl-cysteine for IM-1 incubating under anaerobic conditions at 37 °C for 24 h in an anaerobic DWS MC500 Workstation (Don Whitley Scientific, Bingley, UK).

### 2.2. Pathogen Strains and Preparation

The pathogens used in this study were *Salmonella Typhimurium* BAA-1673 and *Escherichia coli* ATCC53503. For bacterial preparation, BAA-1673 and ATCC53503 strains were cultured in nutrient broth (NB) (Merck KGaA, Darmstadt, Germany) under aerobic conditions at 200 rpm and 37 °C for 24 h. Bacterial cells were harvested by centrifugation at 12,000× *g* for 10 min, washed three times with phosphate-buffered saline (PBS), and resuspended in sterile PBS. The bacterial concentration was adjusted to approximately 1 × 10^8^ CFU/mL using a turbidimeter (Grant Instruments, Cambridge, Royston, UK). The standardized bacterial suspension was used to create a dilution bank for each probiotic strain in sterile PBS to obtain concentrations of 100 CFU/mL and 10 CFU/mL.

### 2.3. Simulated PIF Contamination Assays

A rice-protein-based infant formula (0–6 months) for babies with allergy or intolerance to cow milk protein was prepared according to the manufacturer’s instructions. The complete list of ingredients of the formula is included as Supplementary Materials (Table S1). Once the formula was reconstituted, probiotics and pathogens were added according to the experimental group. Probiotics *B. infantis* IM-1 and *L. rhamnosus* HN001 were added at 10^7^ CFU/mL of reconstituted PIF, and the pathogen was added at 1 or 10 CFU/10 g of reconstituted PIF. Inoculum level of 1 CFU/10 g was used because this represents the lowest theoretical inoculum level that can be introduced into a 10 g sample and subsequently detected using standard enrichment-based protocols. A concentration of 10 CFU/10 g was also used as a one-order-of-magnitude increase, to assess whether increasing the initial pathogen concentration could result in detectable differences or effects under the experimental conditions.

The following experimental groups were established:

Group C0: Rice-based infant formula without probiotics and without *E. coli* or *S. enterica*.
Group C1: Rice-based infant formula without probiotics + *E. coli* or *S. enterica* 1 CFU/10 g of reconstituted PIF.
Group C10: Rice-based infant formula without probiotics + *E. coli* or *S. enterica* 10 CFU/10 g of reconstituted PIF.
Group 1A: Rice-based infant formula supplemented with both probiotics and inoculated with *E. coli* or *S. enterica* at 1 CFU/10 g of reconstituted PIF (Replicate A).
Group 1B: Rice-based infant formula supplemented with both probiotics and inoculated with *E. coli* or *S. enterica* at 1 CFU/10 g of reconstituted PIF (Replicate B).
Group 10A: Rice-based infant formula supplemented with both probiotics and inoculated with *E. coli* or *S. enterica* at 10 CFU/10 g of reconstituted PIF (Replicate A).
Group 10B: Rice-based infant formula supplemented with both probiotics and inoculated with *E. coli* or *S. enterica* at 10 CFU/10 g of reconstituted PIF (Replicate B).

For the probiotic-supplemented conditions, A and B corresponded to independently prepared experimental replicates and not to technical replicates.

Samples were intentionally contaminated with low doses of *E. coli* or *S. enterica* (1 or 10 CFU/10 g of reconstituted PIF) to simulate low levels of contamination. Four independent experiments (E1, E2, E3 and E6) were conducted for the *E. coli* challenge, and four independent experiments (E1, E2, E4 and E5) were performed for the *S. enterica* challenge. In each experiment, the seven experimental groups were evaluated at T0h and T18h, reaching 56 total samples for each pathogen.

Each experimental group was incubated under anaerobic conditions at 37 °C for 18 h (T18h) in an anaerobic DWS MC500 Workstation (Don Whitley Scientific, Bingley, UK). Samples were collected at 0 baseline (T0h) and after 18 h of incubation (T18h), the latter representing the prolonged-storage condition evaluated in this study. The experimental conditions were considered a stringent model in which the potential inhibitory effect of the probiotic combination could be evaluated under conditions that were, a priori, favorable to pathogen proliferation.

### 2.4. Microbial Composition Analysis

Microbial composition was determined by Next-Generation Sequencing (NGS) targeting the V4 region of the bacterial 16S rRNA gene, followed by bioinformatic analysis using QIIME2 and the SILVA database as previously described by the authors [18].

#### 2.4.1. DNA Extraction

Genomic bacterial DNA was extracted from formula using the Milk Bacterial DNA Isolation kit (Norgen Biotek, Thorold ,ON, Canada) following the manufacturer’s instructions. DNA purity, integrity, and quantification was performed with a Nanodrop2000 spectrophotometer (ThermoFisher Scientific, Waltham, MA, USA). A process blank (negative control) and a known bacterial community (positive control) were included in each extraction batch.

#### 2.4.2. Library Preparation, Partial Sequencing of the 16S rRNA Gene and Taxonomic Assignment

The V4 region of the bacterial 16S rRNA gene was amplified by PCR using Tag-Fusion primers based on the 515F-806 primer pair (Table 1). These primers included, at their 5′ ends, the adapter sequences required for the Ion Torrent sequencing workflow and a unique 10-base barcode tag to allow sample identification [19,20,21,22].

Amplicon concentration and size were assessed by microfluidic electrophoresis on a Bioanalyzer (Agilent Technologies, Santa Clara, CA, USA). Individual libraries were subsequently pooled in equimolar amounts and sequenced using the GeneStudio S5 system (ThermoFisher Scientific, Waltham, MA, USA). A mock microbial community of known composition was included as a positive control.

Sequence data were processed using QIIME2 (qiime2-amplicon-2023.9). Reads were quality filtered and denoised using DADA2 to generate amplicon sequence variants (ASVs). Taxonomic assignment was performed using a naïve Bayes classifier trained against the SILVA database. Relative taxonomic abundances were calculated from the resulting ASV table and summarized at the genus level.

Alpha diversity was assessed using the Shannon diversity index and Chao1 richness estimator. For each valid paired observation, changes during incubation were expressed as Δ = T18h − T0h. Alpha-diversity results were analyzed descriptively, and no inferential statistical tests were applied to these metrics; they are presented in the Supplementary Materials.

For visualization of overall community composition, relative abundances were averaged across the valid observations within each experimental condition and time point. In probiotic-supplemented conditions, A and B represented independent replicate preparations of the same experimental condition and were therefore displayed together within each challenge level.

For target-taxon analyses in probiotic-supplemented conditions, T0h and T18h relative abundances were compared using a two-sided paired Wilcoxon signed-rank test. A and B replicate preparations were analyzed jointly within each challenge level while retaining each preparation as an independent paired observation. For *E. coli*, the analysis included eight paired observations for each challenge level (A and B from E1, E2, E3 and E6). For *S. enterica*, the 1 CFU/10 g condition included seven paired observations because Se.-E1-T0h-1A did not yield a valid 16S profile, whereas the 10 CFU/10 g condition included eight paired observations.

For the *S. enterica* challenge assays, the sequence associated with the inoculated organism was not resolved to the genus *Salmonella* by the taxonomic classifier and was initially assigned as *Enterobacteriaceae_A_725029*. Sequence comparison by BLAST 2.17 showed 100% sequence identity with *Salmonella*; considering the known experimental inoculation, this taxon was referred to as *Salmonella* in downstream visualizations and results.

Quality-control samples, including the process blank and mock microbial community, yielded results within the expected ranges [23,24].

## 3. Results

### 3.1. Assays with PIF Reconstituted and Challenged with Low Inoculum Levels of Escherichia coli

Across the four independent assays (E1, E2, E3 and E6), the bacterial community followed markedly different trajectories depending on the experimental condition. In the non-inoculated C0 condition, *Escherichia* remained at low relative abundance throughout incubation. In contrast, in the inoculated non-supplemented conditions C1 and C10, the relative abundance of *Escherichia* markedly increased between T0h and T18h in all assays with paired observations. At T18h, *Escherichia* accounted for 22.2–96.9% and 16.2–98.3% of the bacterial community in C1 and C10, respectively (Figure 1). The magnitude of this increase varied among independent assays, but the overall direction of the change was consistent.

A contrasting pattern was observed in the probiotic-supplemented conditions (1 and 10). A and B represented independent replicate preparations of the same experimental condition and were therefore analyzed jointly within each challenge level. *Escherichia* relative abundance was significantly lower at T18h than at T0h both in the 1 CFU/10 g condition (*n* = 8 paired observations; Wilcoxon signed-rank test, *p* = 0.0156) and in the 10 CFU/10 g condition (*n* = 8; *p* = 0.0078). At T18h, *Escherichia* was either not detected by the 16S sequencing workflow or remained at very low relative abundance (Figure 1).

Analysis of the overall microbial community showed that these differences in *Escherichia* relative abundance occurred within broader changes in community composition. In C1 and C10, the increase in *Escherichia* was accompanied by a shift from communities largely dominated by *Bacillota_I* and members of the *Enterococcaceae* family towards profiles with a high relative representation of *Pseudomonadota* and *Escherichia*. In contrast, probiotic-supplemented samples showed a markedly different community profile at T18h, characterized by the predominance of *Lacticaseibacillus* and *Lactobacillus*. Together, these two genera accounted for 82.8–99.7% of the bacterial community across the probiotic-supplemented samples at T18h, although their relative contributions varied among independent assays and experimental conditions (Figure 2).

Changes in bacterial community composition during incubation were also accompanied by changes in alpha diversity. Shannon diversity increased consistently in C1 and C10, whereas changes in the non-inoculated C0 condition were more variable. In the probiotic-supplemented conditions, Shannon diversity generally decreased between T0h and T18h. Chao1 richness decreased consistently in C10 and across the probiotic-supplemented conditions, whereas a more heterogeneous response was observed in C0 and C1 (Figure S1).

Across the four independent assays, probiotic supplementation was associated with a consistently distinct bacterial community composition after 18 h of incubation compared with non-supplemented conditions.

### 3.2. Assays with PIF Reconstituted and Challenged with Low Inoculum Levels of Salmonella enterica

Across the four independent assays, the bacterial community followed markedly different trajectories depending on the experimental condition. In the non-inoculated C0 condition, the relative abundance assigned to *Salmonella* remained comparatively stable between T0h and T18h, although some inter-assay variability was observed. In contrast, in the inoculated non-supplemented conditions C1 and C10, the relative abundance of *Salmonella* markedly increased between T0h and T18h in all assays, reaching 37.2–97.0% and 93.7–98.3% of the bacterial community at T18h, respectively (Figure 3).

A contrasting pattern was observed in the probiotic-supplemented conditions (1 and 10). *Salmonella* was detected at low relative abundance at T0h and remained at very low relative abundance at T18h across the evaluable samples (Figure 3). When A and B independent replicate preparations were analyzed jointly within each challenge level, the change between T0h and T18h was not statistically significant at 1 CFU/10 g (*n* = 7 paired observations; two-sided Wilcoxon signed-rank test, *p* = 0.1875), whereas a significant decrease was observed at 10 CFU/10 g (*n* = 8; *p* = 0.0078).

Analysis of the overall bacterial community showed that these changes in *Salmonella* relative abundance occurred within broader changes in community composition. In C1 and C10, the increase in *Salmonella* was accompanied by a marked reduction in the relative representation of the initially predominant *Bacillota_I* taxa, particularly members of the *Enterococcaceae* family. In contrast, probiotic-supplemented samples showed a markedly different community profile at T18h, characterized by the predominance of *Lacticaseibacillus* and *Lactobacillus*. Together, these two genera accounted for 91.9–99.1% of the bacterial community across probiotic-supplemented samples at T18h, while *Bifidobacterium* represented a smaller component (0.3–7.1%) (Figure 4).

Changes in bacterial community composition during incubation were also accompanied by changes in alpha diversity. Shannon diversity showed a variable response in C0 and C1, whereas it decreased consistently in C10 and in probiotic-supplemented condition 1. A similar decrease was observed in condition 10 in most assays. Chao1 richness decreased consistently in C1, C10, and across both probiotic-supplemented conditions, while changes in C0 were more variable (Figure S2).

Across the four independent assays, bacterial community composition differed consistently between non-supplemented and probiotic-supplemented conditions during the 18 h incubation period.

## 4. Discussion

The results clearly show that in formulas without probiotics (controls C1 and C10), both *Escherichia coli* and *Salmonella enterica* showed a substantial growth, reaching dominance levels above 90% at T18h even when the contamination was performed using low levels of the pathogen. This was an expected result, because the microorganism growth conditions employed in the study inherently favor the growth of the pathogens *E. coli* and *S. enterica*, which exhibit more rapid growth than the probiotic strains belonging to the genera *Lactobacillus* and *Bifidobacterium* used in this study. Therefore, the experimental conditions were considered a stringent model in which the potential inhibitory effect of the probiotic combination could be evaluated under conditions that were, a priori, favorable to pathogen proliferation, but these conditions should not be interpreted as representative of typical consumer preparation or storage practices.

Formulas supplemented with *B. infantis* and *L. rhamnosus* showed a marked pathogen reduction. In most conditions, pathogens were either undetectable or reduced to <1% of the total microbiota. Furthermore, sequencing revealed a concurrent increase in the genera *Lacticaseibacillus*, *Bifidobacterium* and *Lactobacillus*, which became the majority at T18h, demonstrating effective competition for nutrients and ecological niches.

These findings indicate that the presence of *B. infantis* IM-1 and *L. rhamnosus* HN001 in rice-protein-based infant formula effectively suppresses the proliferation of the pathogen *Escherichia coli* and *Salmonella enterica* within the formula matrix and can contribute to the improvement of the microbiological stability and safety of the infant formula. The observed inhibition of pathogen proliferation may be attributed to several mechanisms:

- Effective competition for nutrients: *B. infantis* IM-1 and *L. rhamnosus* HN001 compete effectively against *E. coli* and *Salmonella enterica* for nutrients in infant formula. Several studies have shown that certain probiotic strains belonging to the genera *Bifidobacterium* and *Lactobacillus* can inhibit the growth of pathogenic microorganisms in infant formula, particularly at storage temperatures ranging from 22 °C to 37 °C [25].
- Antibacterial activity: previous studies have shown that IM-1 and HN001 have antimicrobial activity against *E. coli* and *Salmonella enterica* [26,27,28,29].

The probiotic strain *Bifidobacterium longum* subsp. *infantis* IM-1 has demonstrated antimicrobial activity against *Escherichia coli*, *Cronobacter sakazakii*, *Listeria monocytogenes*, and *Clostridium difficile* in co-culture experiments. Adhesion assays performed using the intestinal epithelial cell line HT-29 showed that IM-1 could displace certain pathogens from the enterocyte layer, particularly *C. sakazakii* and *Salmonella enterica*, preventing the adhesion of *C. sakazakii* and *Shigella sonnei* [27]. Human safety and tolerability studies conducted in both adults and infants using infant formulas containing the probiotic strain *Bifidobacterium longum* subsp. *infantis* IM-1 have consistently demonstrated good tolerability, with no adverse effects reported in any of the clinical trials performed [30,31,32,33,34,35]. In vivo studies have also demonstrated that *Bifidobacterium longum* subsp. *infantis* IM-1 can colonize the intestinal tract [36]. Furthermore, the cell-free supernatant of IM-1 exerted a protective effect against cytotoxicity induced by *Escherichia coli* CECT 515 in Caco-2 cell cultures, restoring cell viability to levels comparable with untreated controls. Both *Bifidobacterium longum* subsp. *infantis* IM-1 and its supernatant reduced the secretion of pro-inflammatory cytokines by Caco-2 cells and co-cultures exposed to *E. coli* CECT 515, although the response was less pronounced in co-culture systems, suggesting modulation by dendritic cells. Overall, these findings support the immunomodulatory activity of this probiotic strain, which may underlie its previously reported beneficial effects [37]. Studies conducted in early-weaned piglet models have demonstrated that *Bifidobacterium longum* subsp. *infantis* IM-1 exhibits activity against enterotoxigenic *Escherichia coli* F4 and *Salmonella Typhimurium*, reducing fecal pathogen loads and improving intestinal health following oral challenge [38,39,40,41]. In these studies, *Bifidobacterium longum* subsp. *infantis* IM-1 reduced diarrhea scores in infected animals, improved fermentation profiles, stimulated intestinal immune responses by increasing villus intraepithelial lymphocyte counts, and enhanced the villus-to-crypt ratio.

*Lacticaseibacillus rhamnosus* HN001 is a probiotic strain that has demonstrated safety in clinical studies involving infants [42,43]. Its consumption has been associated with increased levels of beneficial intestinal bacteria in children [42,44], promotion of immune system development and homeostasis throughout infancy and childhood [42,43,45,46,47], immunostimulatory activity [28], and antibacterial activity against several gastrointestinal pathogens, including *Salmonella*, *E. coli*, *Candida*, and *Streptococcus* species [41,48,49,50,51,52,53,54].

The inclusion of probiotics does not replace appropriate infant-formula preparation practices; however, it may provide an additional margin of safety against common errors occurring during home preparation. This could translate into a reduced risk of infection in infants and an improvement in the microbiological quality of infant formula products, although further studies would be required to demonstrate this. The studies cited in the previous section demonstrate that, despite preventive preparation instructions mandated by national health authorities on infant formula labeling, the proportion of parents and caregivers who continue to prepare infant formula incorrectly remains substantial. In practice, many parents do not reach the recommended preparation temperature of 70 °C, thereby allowing probiotic survival and, potentially, the exertion of their inhibitory effects against pathogens. When bottles are prepared at temperatures below 70 °C, pathogens such as *Cronobacter sakazakii* may survive and proliferate [25,55,56]. Probiotics contained in infant formula lose viability or are inactivated when reconstituted at 70 °C; however, they survive at lower temperatures (e.g., 50–60 °C) [56,57,58,59,60].

The addition of probiotics such as IM-1 and HN001 in infant formula significantly reduced the proliferation of *Escherichia coli* and *Salmonella enterica* under the experimental conditions evaluated and could potentially contribute to reducing the risks for infants who may consume improperly prepared formula. The rationale for this approach is that these probiotic strains may help reduce the proliferation of pathogens when feeding bottles are not adequately heated and fail to reach the 70 °C temperature recommended by health authorities. The presence of probiotics does not eliminate the need for appropriate hygienic practices, nor does it replace the recommendations issued by health authorities to reconstitute infant formula at 70 °C, but it could potentially reduce the risk of pathogen infection [55,56].

The present study was conducted under controlled experimental conditions and evaluated pathogen behavior in an in vitro model. Therefore, the findings should not be interpreted as evidence of improved product safety, reduced infection risk, or clinical protection. Further studies, including industrial-scale validation and in vivo assessments, are needed to confirm the practical implications of these observations.

## 5. Conclusions

In conclusion, the probiotic combination of IM-1 and HN001 exerts a robust inhibitory effect by limiting the proliferation of *E. coli* and *S. enterica* in infant formulas contaminated at low-level concentrations with these pathogens and may potentially provide an additional restrictive barrier against pathogens in reconstituted PIF. While probiotics do not replace the need to prepare infant formula with water at 70 °C, they could provide an additional layer of safety in cases of domestic preparation errors, where probiotics may remain viable (up to approximately 60 °C) while pathogens may otherwise proliferate. The incorporation of these strains could potentially have a positive public health impact by contributing to the microbiological stability and quality of infant nutrition products, particularly in regions with high rates of suboptimal preparation practices, but, as we mentioned before, does not replace the need to prepare infant formula with water at 70 °C.

## Figures and Tables

**Figure 1:**
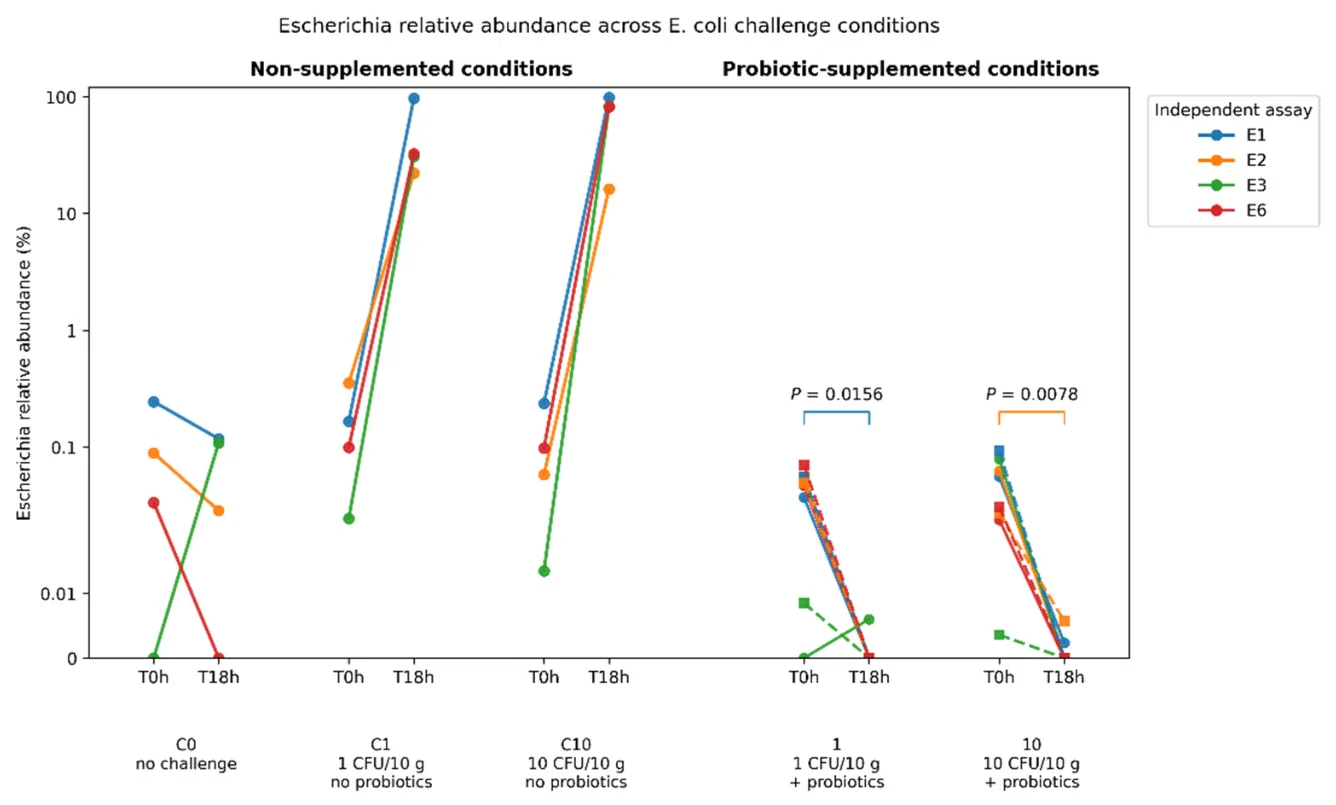
Relative abundance of *Escherichia* across *E. coli* challenge conditions at T0h and T18h. Results are shown for the four included independent assays (E1, E2, E3 and E6). C0 corresponds to the non-inoculated, non-supplemented control; C1 and C10 correspond to non-supplemented formula challenged with 1 or 10 CFU/10 g, respectively; and conditions 1 and 10 correspond to probiotic-supplemented formula challenged with 1 or 10 CFU/10 g, respectively. For the probiotic-supplemented conditions, A and B were independent replicate preparations of the same experimental condition and are therefore displayed together; solid and dashed lines distinguish between A and B replicates, respectively. Colors identify the independent assays consistently across conditions. The y-axis is displayed using a symmetric logarithmic transformation to allow visualization of both very low and high relative-abundance values; consequently, the spacing between y-axis tick marks is not linear. For probiotic-supplemented conditions 1 and 10, T0h and T18h values were compared using a two-sided paired Wilcoxon signed-rank test after pooling the independent A and B replicate preparations within each challenge level. Exact *p*-values are indicated above the corresponding comparisons.

**Figure 2:**
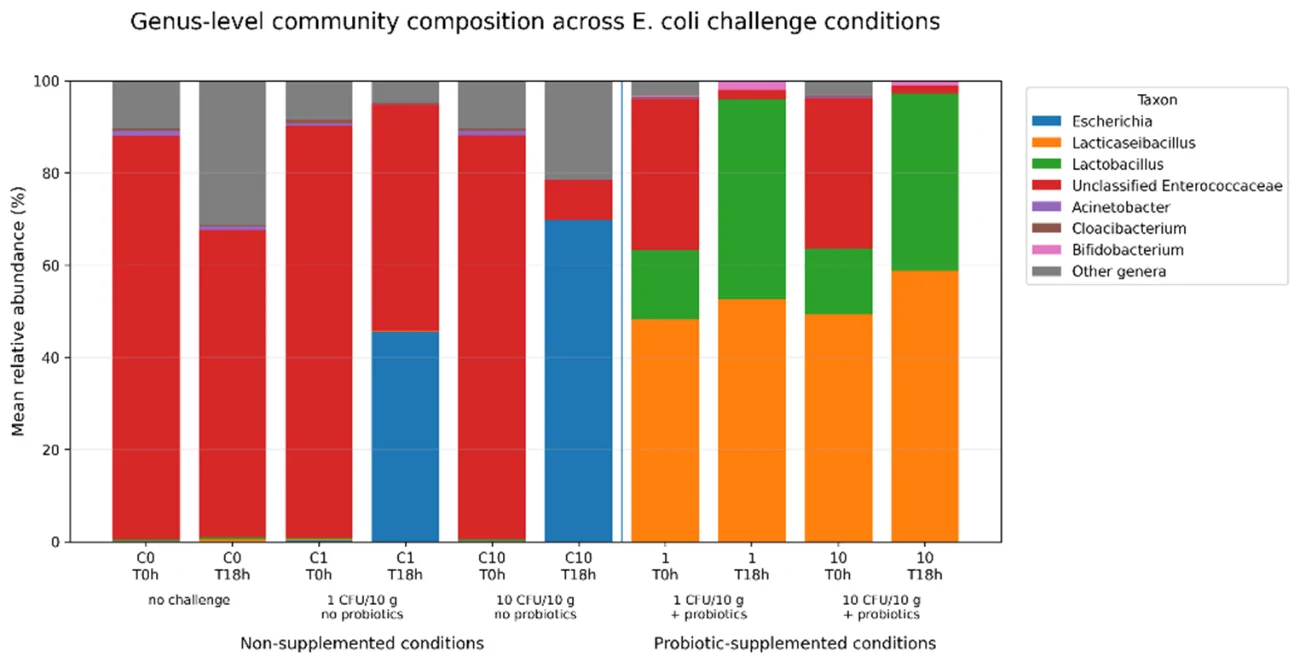
Genus-level community composition across the *E. coli* challenge conditions. Stacked bar plots show the mean relative abundance of the dominant bacterial genera at T0h and T18h across the four included independent assays (E1, E2, E3 and E6). Non-supplemented conditions are shown on the left (C0, C1 and C10), whereas probiotic-supplemented conditions are shown on the right (1 and 10). For the probiotic-supplemented conditions, A and B corresponded to independent replicate preparations of the same challenge condition and were therefore pooled within each challenge level. Thus, C0, C1 and C10 represent the mean of four observations per time point, whereas conditions 1 and 10 represent the mean of eight observations per time point. Selected dominant genera are displayed individually, while the remaining taxa are grouped as *Other genera*.

**Figure 3:**
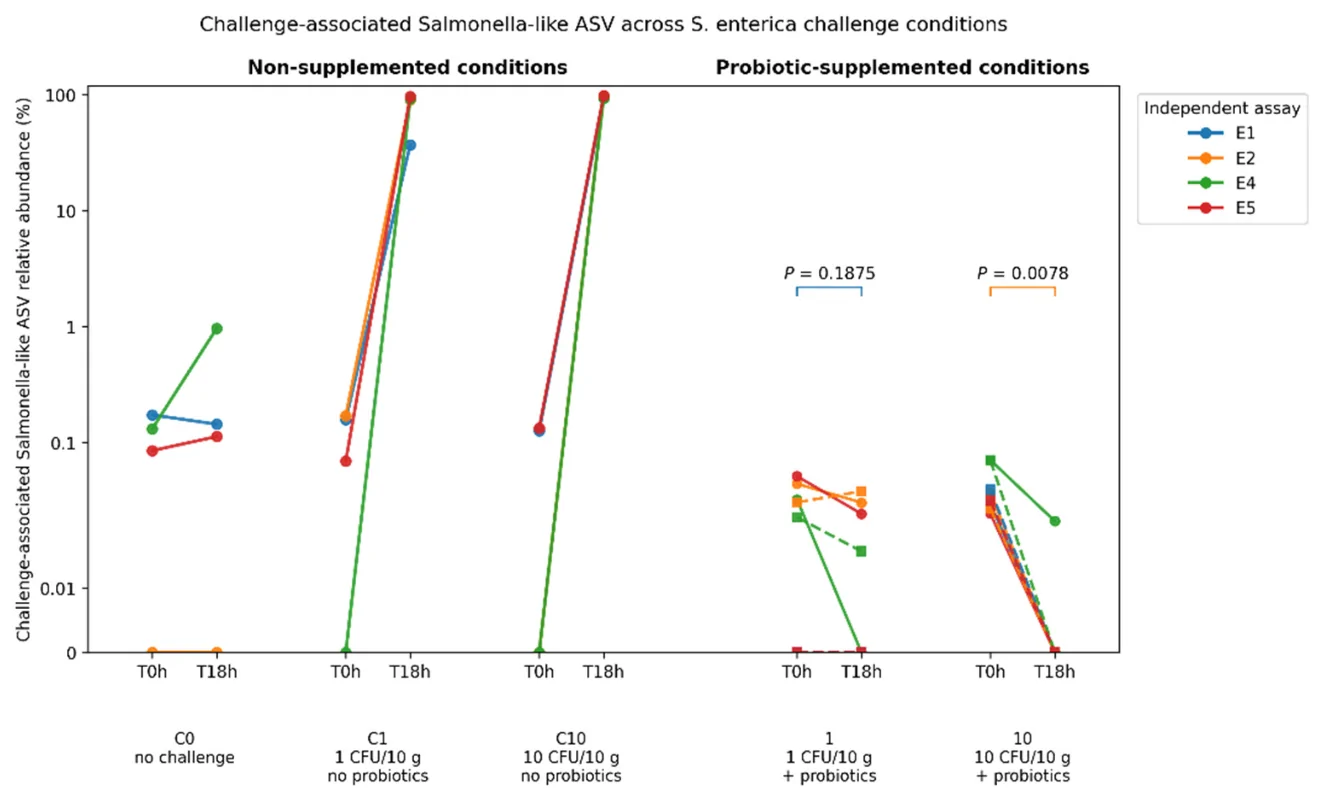
Relative abundance of *Salmonella* across *S. enterica* challenge conditions at T0h and T18h. Results are shown for the four independent assays (E1, E2, E4 and E5). C0 corresponds to the non-inoculated, non-supplemented control; C1 and C10 correspond to non-supplemented formula challenged with 1 or 10 CFU/10 g, respectively; and conditions 1 and 10 correspond to probiotic-supplemented formula challenged with 1 or 10 CFU/10 g, respectively. For the probiotic-supplemented conditions, A and B were independent replicate preparations of the same experimental condition and are displayed together; solid and dashed lines distinguish between A and B replicates, respectively. Colors identify the independent assays consistently across conditions. The y-axis is displayed using a symmetric logarithmic transformation to allow visualization of both very low and high relative-abundance values. T0h and T18h values were compared using a two-sided paired Wilcoxon signed-rank test with A and B replicate preparations analyzed jointly within each challenge level. Exact *p*-values are indicated above the corresponding comparisons. Condition 1 included seven paired observations because one T0h sample did not yield a valid 16S profile; condition 10 included eight paired observations.

**Figure 4:**
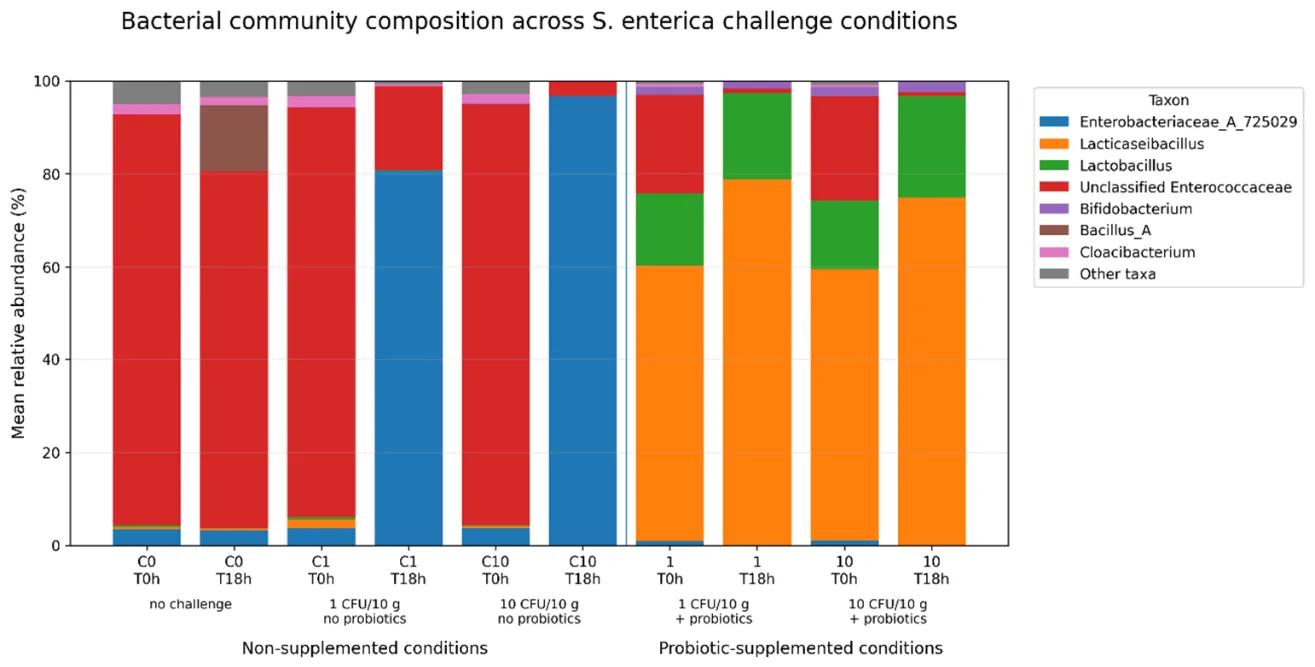
Bacterial community composition across the *S. enterica* challenge conditions. Stacked bar plots show the mean relative abundance of the dominant bacterial taxa at T0h and T18h across the four independent assays (E1, E2, E4 and E5). Non-supplemented conditions are shown on the left (C0, C1 and C10), whereas probiotic-supplemented conditions are shown on the right (1 and 10). For the probiotic-supplemented conditions, A and B corresponded to independent replicate preparations of the same challenge condition and were therefore pooled within each challenge level. Thus, C0, C1 and C10 represent the mean of four observations per time point; condition 10 represents the mean of eight observations per time point; and condition 1 represents seven observations at T0h and eight at T18h because one T0h sample did not yield a valid 16S profile. Selected dominant taxa are displayed individually, while the remaining taxa are grouped as *Other taxa*.

**Table 1:** Fusion-tag primer sequences used to amplify the V4 16S rRNA gene regions. * A unique barcode was used for each sample.

Name and Direction	Adaptor	Key	Barcode	Spacer	Target 16S Primer
V4_forward	CCATCTCATCCCTGCGTGTCTCCGAC	TCAG	CTAAGGTAAC	GAT	GTGCCAGCMGCCGCGGTAA
V4_reverse	CCTCTCTATGGGCAGTCGGTGAT		*	CC	GGACTACHVGGGTWTCTAAT

## Data Availability

The data presented in this study are available on request from the corresponding author. The data are not publicly available due to privacy restrictions.

## Supplementary Materials

The following supporting information can be downloaded at: https://www.mdpi.com/article/10.3390/foods15193477/s1, Figure S1: Shannon diversity (A) and Chao1 richness (B) from T0 to T18 across independent *E. coli* assays; Figure S2: Shannon diversity (A) and Chao1 richness (B) from T0 to T18 across independent *Salmonella enterica* assays; Table S1: Detailed nutritional composition of the rice protein-based infant formula used in the experimental assays.

## Abbreviations

The following abbreviations are used in this manuscript:

PIF	Powdered Infant Formula
WHO	World Health Organization
MRS	Man, Rogosa and Sharpe
NB	Nutrient Broth
CFU	Colony-Forming Unit
NGS	Next-Generation Sequencing
DNA	Deoxyribonucleic Acid
BLAST	Basic Local Alignment Search Tool
PCR	Polymerase Chain Reaction
