# Supplementary material for: Probiotics as a Barrier Against Salmonella enterica and Escherichia coli in Contaminated Rice-Protein-Based Infant Formulas
Source: Foods. 2026 Sep 28;15(19):3477. doi: 10.3390/foods15193477 (PMC13664493; doi:10.3390/foods15193477)
Supplement: Supplementary file 1 [file foods-15-03477-s001.zip › foods-4503180-supplementary.pdf]

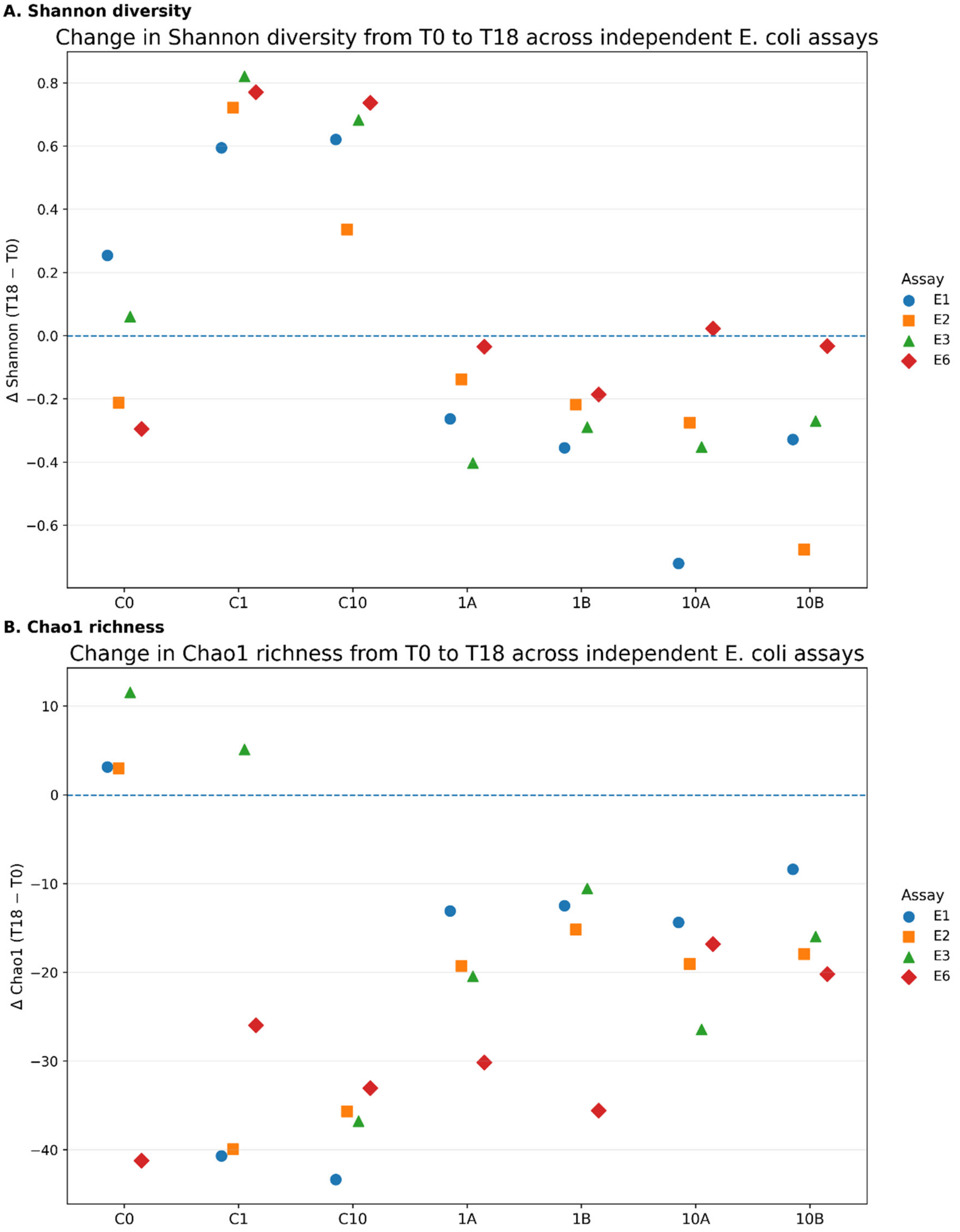


**Figure S1.** Shannon diversity (**A**) and Chao1 richness (**B**) from T0 to T18 across independent *E. coli* assays.

A. Shannon diversity

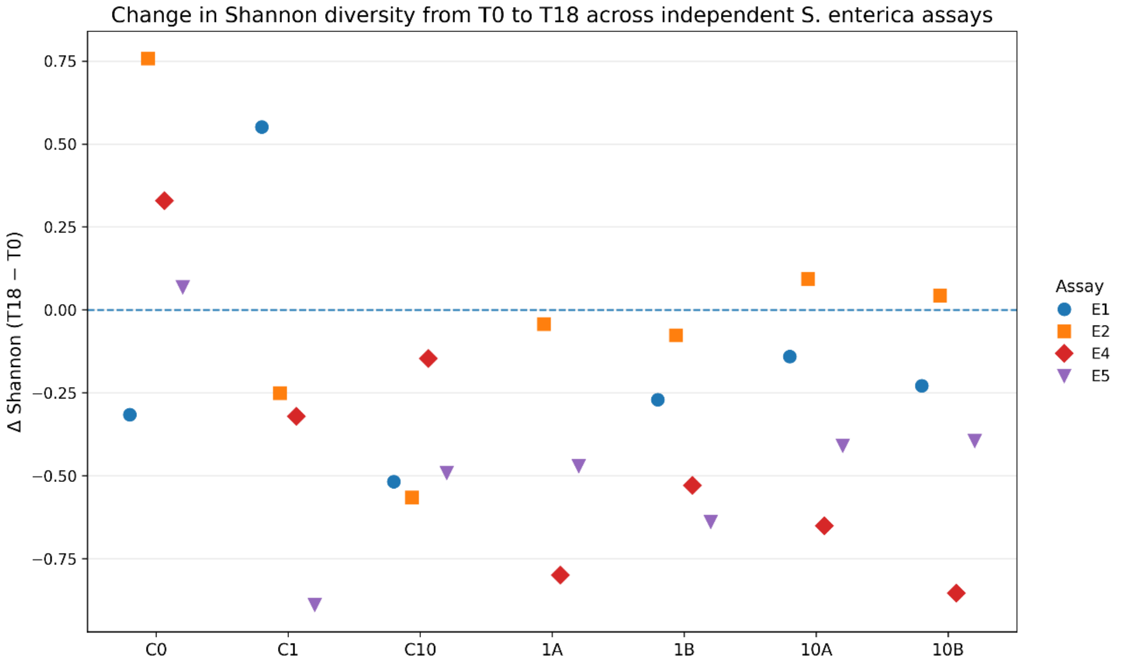


B. Chao1 richness

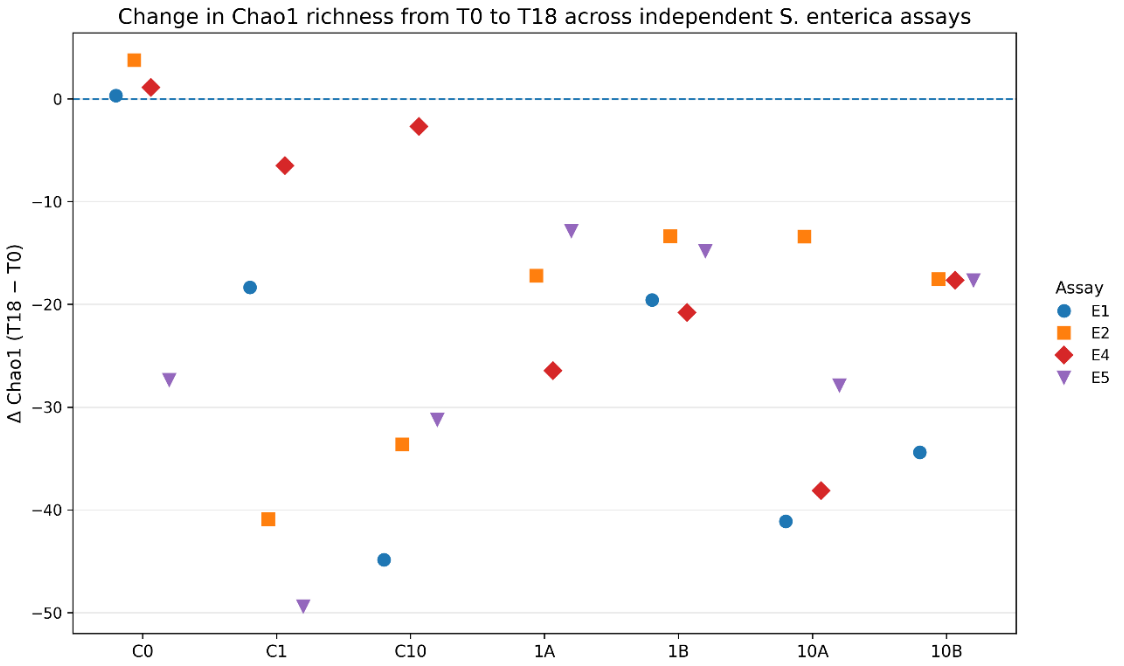


**Figure S2.** Shannon diversity (**A**) and Chao1 richness (**B**) from T0 to T18 across independent *Salmonella enterica* assays.

**Table S1.** Detailed nutritional composition of the rice protein-based infant formula used in the experimental assays. Energy, macronutrients, selected bioactive compounds, vitamins, and minerals are reported per 100 g of powder, per 100 mL of reconstituted formula (13.5% w/v dilution), and per 100 kcal. Abbreviations: MCT, medium-chain triglycerides; AA, arachidonic acid; DHA, docosahexaenoic acid. Nutritional information was obtained from the manufacturer's specifications.

| | UNITS | 100 g POWDER | 100 ml DIL. 13,5% | 100 kcal |
|---|---|---|---|---|
| **ENERGY** | kJ | 2074 | 280 | |
| | kcal | 496 | 67 | |
| **FAT** | g | 25,5 | 3,4 | 5,1 |
| SATURATED FATTY ACIDS | g | 13,8 | 1,9 | 2,8 |
| MONOUNSATURATED FATTY ACIDS | g | 6,8 | 0,9 | 1,4 |
| POLIUNSATURATED FATTY ACIDS | g | 4,9 | 0,7 | 1,0 |
| MCT | g | 6,6 | 0,9 | 1,3 |
| LINOLEIC ACID | mg | 2640 | 356 | 532 |
| a-LINOLENIC ACID | mg | 298 | 40 | 60,1 |
| AA ACID | mg | 125 | 16,9 | 25,2 |
| DHA ACID | mg | 125 | 16,9 | 25,2 |
| **CARBOHYDRATES** | g | 51,8 | 7,00 | 10,4 |
| MALTODEXTRINE | g | 39,8 | 5,37 | 8,0 |
| CORN STARCH | g | 12,0 | 1,62 | 2,42 |
| **FIBER** | g | 5,7 | 0,8 | 1,2 |
| FRUCTOOLIGOSACCHARIDES | g | 2,85 | 0,4 | 0,6 |
| INULIN | g | 2,85 | 0,4 | 0,6 |
| **PROTEINS** | g | 12,0 | 1,6 | 2,4 |
| **NUCLEOTIDES** | mg | 20,5 | 2,8 | 4,1 |
| TAURINE | mg | 35 | 4,7 | 7,1 |
| CHOLINE | mg | 150 | 20,3 | 30,2 |
| INOSITOL | mg | 25 | 3,4 | 5,0 |
| L-CARNITINE | mg | 7 | 1,0 | 1,4 |
| GANGLIOSIDOS | mg | 6,37 | 0,86 | 1,28 |

| **VITAMINS** | UNITS | 100 g POWDER | 100 ml DIL. 13,5% | 100 kcal |
|---|---|---|---|---|
| VITAMIN A | µg | 440 | 59 | 89 |
| VITAMIN D | µg | 11,0 | 1,5 | 2,2 |
| VITAMIN E | mg | 9,0 | 1,2 | 1,8 |
| VITAMIN K | µg | 40,0 | 5,4 | 8,1 |
| VITAMIN B1 | µg | 770 | 104 | 155,2 |
| VITAMIN B2 | µg | 1150 | 155 | 232 |
| VITAMIN B6 | µg | 770 | 104 | 155,2 |
| VITAMIN B12 | µg | 1,4 | 0,19 | 0,3 |
| VITAMIN C | mg | 70 | 9,5 | 14,1 |
| FOLIC ACID | µg | 49,8 | 6,7 | 10,0 |
| PANTOTHENIC ACID | mg | 3,2 | 0,4 | 0,6 |
| NIACINE | mg | 5 | 0,7 | 1,0 |
| BIOTINE | µg | 12 | 1,6 | 2,4 |

| **MINERALS** | UNITS | 100 g POWDER | 100 ml DIL. 13,5% | 100 kcal |
|---|---|---|---|---|

| | | | | |
|---|---|---|---|---|
| SODIUM | mg | 225 | 30 | 45 |
| POTASSIUM | mg | 500 | 68 | 101 |
| CHLORINE | mg | 420 | 57 | 85 |
| CALCIUM | mg | 650 | 88 | 131 |
| PHOSPHOROUS | mg | 395 | 53 | 80 |
| IRON | mg | 5 | 0,7 | 1,0 |
| MAGNESIUM | mg | 45 | 6,1 | 9,1 |
| ZINC | mg | 7,5 | 1,0 | 1,51 |
| COPPER | µg | 370 | 50 | 75 |
| IODINE | µg | 100 | 13,5 | 20,2 |
| MANGANESE | µg | 150 | 20,3 | 30,2 |
| SELENIUM | µg | 20 | 2,7 | 4,0 |
| CROME | µg | 20 | 2,7 | 4,0 |
| MOLIBDENE | µg | 30 | 4,1 | 6,0 |
| FLUORIDE | µg | 100,0 | 13,5 | 20,2 |
